# Epileptiform activity in brain organoids derived from patient with Glucose Transporter 1 Deficiency Syndrome

**DOI:** 10.3389/fnins.2024.1498801

**Published:** 2024-11-13

**Authors:** Y. Müller, L. Lengacher, F. Friscourt, C. Quairiaux, L. Stoppini, P. J. Magistretti, S. Lengacher, C. Finsterwald

**Affiliations:** ^1^GliaPharm SA, Geneva, Switzerland; ^2^Functional Brain Mapping Lab, Department of Basic Neuroscience, University of Geneva, Geneva, Switzerland; ^3^Neurosurgery Clinic, Department of Clinical Neuroscience, University Hospital Geneva, Geneva, Switzerland; ^4^Tissue Engineering Laboratory, HEPIA HES-SO University of Applied Sciences and Arts Western Switzerland, Geneva, Switzerland

**Keywords:** GLUT1-DS, epilepsy, brain organoid, astrocyte, glucose, brain energy metabolism, drug development, multielectrode array

## Abstract

**Introduction:**

Glucose Transporter 1-Deficiency Syndrome (GLUT1-DS) is a rare genetic disorder caused by mutations in the gene encoding for GLUT1 and characterized by impaired glucose uptake in the brain. This leads to brain hypometabolism and the development of symptoms that include epilepsy, motor dysfunctions and cognitive impairment. The development of patient-specific *in vitro* models is a valuable tool for understanding the pathophysiology of rare genetic disorders and testing new therapeutic interventions.

**Methods:**

In this study, we generated brain organoids from induced pluripotent stem cells (iPSCs) derived either from a GLUT1-DS patient or a healthy individual. The functional organoids were analyzed for cellular composition, maturity, and electrophysiological activity using a custom-made microelectrode array (MEA) platform, which allowed for the detection of spikes, burst patterns, and epileptiform discharges.

**Results:**

Immunostaining revealed a similar distribution of neurons and astrocytes in both healthy and GLUT1-DS brain organoids, though GLUT1-DS brain organoids exhibited reduced cellular density and smaller overall size. Electrophysiological recordings demonstrated functional spike profiles in both organoid types. Notably, our study demonstrates that brain organoids derived from a GLUT1-DS patient exhibit distinct epileptiform activity and heightened sensitivity to glucose deprivation, reflecting key features of the disorder.

**Discussion:**

These findings validate the use of brain organoids as a model for studying GLUT1-DS and highlight their potential for testing novel therapeutic strategies aimed at improving glucose metabolism and managing epilepsy in patients.

## Introduction

Glucose Transporter 1-Deficiency Syndrome (GLUT1-DS) is an heterozygous genetic neurodevelopmental disorder caused by mutations in the *SLC2A1* gene, which encodes the glucose transporter 1 (GLUT1) protein. Different types of mutations can affect GLUT1 function through the impairment of its expression, processing or trafficking to the plasma membrane (Leen et al., 2010). Defective GLUT1 results in insufficient glucose supply to the brain, leading to brain hypometabolism and neurological impairments that include motor dysfunctions, cognitive deficit and epilepsy that is refractive to classical antiepileptic treatments (Pearson et al., 2013). The disorder starts manifesting in infancy with seizures being prominent at young age and develops into childhood and adulthood with motor and cognitive dysfunctions (Gras et al., 2014). The syndrome is considered rare, with an estimated prevalence of approximately 1 in 24,000 to 1 in 90,000 globally (Klepper et al., 2020). However, it is believed that the condition is underdiagnosed due to its variable clinical presentation (Lopez-Rivera et al., 2020; Symonds et al., 2019). Diagnosis is typically confirmed through genetic testing for *SLC2A1* mutations, functional assessment of GLUT1 transport capacity in erythrocytes, and identification of abnormally low cerebrospinal fluid (CSF) glucose and lactate levels despite normal blood concentrations (Pascual et al., 2002; Tang and Monani, 2021; Mochel et al., 2023).

The primary treatment for GLUT1-DS is the ketogenic diet, a high-fat, low-carbohydrate diet that mimics the metabolic state of fasting and provides an alternative energy source for neurons in the form of ketone bodies (Klepper et al., 2020; Tang et al., 2019). While the ketogenic diet effectively improves seizure control, adherence is challenging, highlighting the need for novel therapeutic strategies to enhance brain glucose uptake (Tang et al., 2019; Ye et al., 2015). Emerging disease-modifying approaches for GLUT1-DS include gene therapy (Tang et al., 2017) and pharmacological interventions aimed at restoring brain glucose transport and metabolism, including ours (Beard et al., 2021; Kathote et al., 2023; Tang et al., 2019).

In the brain, GLUT1 is predominantly expressed by endothelial cells and astrocytes. Its deficiency severely compromises glucose transport across the blood–brain barrier, diminishing the brain’s energy supply. Astrocytes, with their complex cytoarchitecture, are crucial in sensing and responding to extracellular changes. Their numerous processes form organized anatomical domains, enabling interactions with both synapses and brain capillaries. At synapses, astrocytic processes express glutamate transporters that detect neuronal activity, while their endfeet, in contact with vascular endothelium, express GLUT1. This strategic positioning allows astrocytes to facilitate glucose uptake from the blood circulation. In astrocytes, glucose is primarily metabolized through aerobic glycolysis to produce lactate. Lactate is then shuttled to neurons via specific monocarboxylate transporters (MCTs), namely MCT1 and MCT4 on astrocytes, and MCT2 on neurons (Magistretti and Allaman, 2015). In neurons, lactate is converted into pyruvate, which enters the tricarboxylic acid (TCA) cycle in the mitochondria to produce ATP through oxidative phosphorylation. Transfer of lactate from astrocytes to neurons, which is known as the astrocyte neuron lactate shuttle (ANLS), plays a critical role in neuroprotection and processes such as synaptic plasticity and memory consolidation (Jourdain et al., 2016; Jourdain et al., 2018; Margineanu et al., 2018; Suzuki et al., 2011; Yang et al., 2014; Magistretti and Allaman, 2018). Interestingly, glutamatergic signaling has also been shown to enhance membrane expression of GLUT1 in oligodendrocytes, thereby providing increased metabolic support to axons in response to neuronal activity (Looser et al., 2024; Saab et al., 2016).

Three-dimensional (3D) neural tissue cultures, including brain slices, organotypic cultures, neurospheres and brain organoids offer advanced platforms for studying the complex neuronal activity in multicellular environment underlying both physiological and pathological brain functions (Benito-Kwiecinski and Lancaster, 2020; Chiaradia and Lancaster, 2020; Frega et al., 2014; Stoppini et al., 2024; Sundstrom et al., 2012; Loussert-Fonta et al., 2023). These models, combined with micro-electrode array (MEA) technology, allow for non-invasive monitoring of electrophysiological activity across neural networks (Forro et al., 2021; Steidl et al., 2006; Stett et al., 2003). MEA technology enables precise mapping of neuronal communication, as single spikes can be detected across multiple electrodes, providing a detailed map of functional interactions within a neuronal population (Pelkonen et al., 2021). This capability makes MEA technology ideal for studying neural network dysfunctions in disorders like GLUT1-DS. However, a major challenge in brain organoid culture is the extended time required for neural circuits to develop sufficiently for MEA recording. The air-liquid interface (ALI) technique, where organoids are grown on a porous membrane with nutrients supplied from below while allowing sufficient oxygenation from above, is a culturing approach that supports long-term tissue maturation and survival (Stoppini et al., 2024).

In this study, we utilized 3D brain organoids derived from induced-pluripotent stem cells (iPSCs) from healthy origin or GLUT1-DS patient, cultured on MEA biochips designed for functional monitoring of neuronal networks at ALI. We recorded electrical signal to characterize epileptiform patterns, providing a platform to test novel therapeutic strategies aimed at restoring proper metabolism in GLUT1-DS and assessing their impact on epileptic features.

## Materials and methods

### Generation of brain organoids from induced pluripotent stem cells

Induced-pluripotent stem cells (iPSCs) were obtained and differentiated into neural stem cells (NSCs), which were subsequently used to generate brain organoids. The iPSCs were purchased from the Corriell Institute and included cells derived from a healthy volunteer (Male, 23YR, GM28404) and from GLUT1-DS patient with an identified mutation in the *SLC2A1* gene (c. 1,454\u00B0C > T (p.Pro485Leu), Female, 19YR, GM27896). After thawing, the iPSCs were cultured on dishes coated overnight with human recombinant laminin isoform 521 (10 μg/mL; Biolamina) diluted in PBS containing 0.9 mM CaCl_2_ + 0.5 mM MgCl_2_. The cells were maintained in mTeSR Plus medium (Stemcell Technologies), with daily medium changes for a period of 7 days until they reached 80–90% confluence. At this stage, cells were harvested using 1x ACCUTASE™ in Dulbecco’s PBS supplemented with 0.5 mM EDTA * 4Na and 3 mg/L Phenol red (Stemcell Technologies) and were either cryopreserved in Cryostor CS10 (Stemcell Technologies) at-196°C or used for differentiation into NSCs according to described procedure (Yan et al., 2013). Briefly, iPSCs were plated in 60 mm dishes coated with Geltrex (Life Technologies) at a seeding density of 40,000 cells/cm^2^ and cultured in Neurobasal Medium (Thermo Fisher Scientific) supplemented with 1x neural induction supplement (Life Technologies) for 7 days. Following neural induction, the resulting NSCs were expanded in a medium composed of a 1:1 mixture of Neurobasal Medium and KnockOut DMEM/F-12 Basal Medium supplemented with 1x GlutaMAX Supplement and 1x Neural Induction supplement (Life Technologies). Cells were grown for 7 days until they reached 70–90% confluence. Cells were then collected using ACCUTASE™ and seeded at a density of 25,000 cells/cm^2^, treated with Y-27632 dihydrochloride Rho kinase inhibitor (5 μM; Abcam) for 24 h. Next, mature NSCs were differentiated into brain organoids following published procedure (Govindan et al., 2020). Briefly, 250,000 NSCs were seeded in a well of 6-well plate with NSC expansion medium (Life Technologies) and grown under constant 80 rpm rotation at 37°C and 5% CO_2_. After one week, when cells started aggregating and forming spheres, the medium was switched to DIFF1 medium, consisting of DMEM/F12 + GlutaMAX, supplemented with 1.8% BSA, 1x Stempro hESC Supplement (Life Technologies), brain-derived neurotrophic factor (BDNF; 20 ng/mL; Life Technologies), glial cell line-derived neurotrophic factor (GDNF; 20 ng/mL; Life Technologies), dibutyryl cyclic AMP (500 μM; Merck) and 2-phospho-ascorbic acid (200 μM; Merck). At the end of the second week, the medium was replaced by DIFF2 medium, a 1:1 mixture of DIFF1 medium and Neuron Differentiation/Maintenance Medium (NDM), composed of Neurobasal Plus medium (Life Technologies) supplemented with 2% B27 Plus supplement (Life Technologies) and 0.25x GlutaMAX-I (Life Technologies). This medium was used for 3 weeks, with weekly changes. Finally, organoids were maintained in NDM medium, which was changed weekly, until they reached cellular maturity at a total of 8 weeks after NSCs.

### Immunocytochemistry and image analysis

Brain organoids were fixed in 4% paraformaldehyde (Thermoscientific) for 45 min, washed with PBS and with a 30% sucrose solution (Merck), followed by overnight incubation in 30% sucrose under rotation at 4°C. After sedimentation, organoids were embedded in Epredia Cryochrome Embedding Resin (Fisher Scientific) and flash-frozen at-80°C. 20 μm-thick slices were cut using a Leica CM3050 S Cryostat and placed on MicroSlides SuperFrost Plus microscopy slides (Menzel Epredia). After storage at-20°C, slices were thawed, tissues circled with hydrophobic barrier pen (Merck), and rehydrated with PBS for 5 min at room temperature. Tissues were permeabilized and blocked with PBS + 0.1% Triton-X100 (Merck) + 5% Bovine Serum Albumin Fraction 5 (BSA; Applichem) for 60 min at room temperature, followed by overnight incubation at 4°C with primary antibodies diluted in PBS + 2% BSA. Primary antibodies consisted in anti-microtubule associated protein 2 (MAP2) antibody from chicken (1/5000, Invitrogen) and anti-glial fibrillary acidic protein (GFAP) antibody from rabbit (1/500, Agilent). Secondary antibodies were diluted in PBS + 2% BSA and incubated for 2 h at room temperature, followed by DAPI staining for 30 min (1 μg/mL; Life Technologies). Secondary antibodies consisted in Alexa Fluor 647 anti-chicken (1/1000; Abcam) and FITC anti-rabbit (1/400; Jackson Immunoresearch). Slices were then dried and mounted with Fluoromount (Merck). Fluorescent images were captured using Nikon AX/AX R confocal microscope system with 20X magnification lens.

### Image processing

For immunostaining analysis, the surface area of each marker was quantified using Image J software (v. 1.53) and expressed relative to total organoid surface area. For cell density analysis, nuclei were counted using Image J software (v. 1.53) and expressed as the number of DAPI-positive cells per mm^2^.

### Real-time quantitative PCR

Real-time quantitative PCR (RT-qPCR) was performed as described previously (Finsterwald et al., 2021). Briefly, RNAs were extracted using the NucleoSpin RNA Plus kit (Macherey-Nagel), and reverse transcription was done with 100 ng of RNA using the High-Capacity RNA-to-cDNA Kit (Life Technologies) following the manufacturers’ instructions. RT-qPCR was performed with the QuantStudio 6 Flex Real-Time PCR System (Applied Biosystems), using the Power UP SYBR Green Master mix (Applied Biosystems), and the following primers: *β*-actin, forward 5’-GCACCCAGCACAATGAAGATCAAG-3′, reverse 5’-TCATACTCCTGCTTGCTGATCCAC-3′; cyclophilin, forward 5’-TCAAGATGTCGCACCCGTC-3′, reverse 5’-TTCCGCAGTTTTGGGTACGA-3′; GLUT1, forward 5′- TGGCATCAACGCTGTCTTCT-3′, reverse 5′- CTAGCGCGATGGTCATGAGT-3′; synaptophysin (SYP), forward 5’-CGAGGTCGAGTTCGAGTACC3’-, reverse 5’-TGACGAGGAGTAGTCCCCAA-3′; GFAP, forward 5’-GTGCAGACCTTCTCCAACCT-3′, reverse 5’-CACCACGATGTTCCTCTTGA-3′; MAP2, forward 5’-AACCGAGGAAGCATTGATTG-3′, reverse 5’-TTCGTTGTGTCGTGTTCTCA-3′; vesicular glutamate transporter 2 (VGLUT2), forward 5-GCTTCTGCATCTCCTTCGGT-3′, reverse 5′- AGCTCCGAAAACCCTGTTGG-‘3’; glutaminase (GLS), forward 5′- CTTTCCCCAAGGACAGGTGG-3′, reverse 5′- TGAGGTGTGTACTGGACTTGG-3′; glutamate decarboxylase 65 (GAD65), forward 5′- TCTCCATGCAACAGACCTGC-3′, reverse 5′- TGCTGTTGATGTCAGCCAGT-3′; glutamate decarboxylase 67 (GAD67), forward 5′- AACTGGGGCTCAAGATCTGC-3′, reverse 5′- CCTGTGCGAACCCCATACTT-3′; 2′,3’-Cyclic nucleotide 3′-phosphodiesterase (CNP), forward 5’-GAAATGGCCGACCAGTACCA-3′, reverse 5’-CTCATCCCCAGGGACGAATTG-3′; OCT4, forward 5’-AACCTGGAGTTTGTGCCAGGGTTT-3′, reverse 5’-TGAACTTCACCTTCCCTCCAACCA-3′; nestin (NES) forward 5’-GGAGAAACAGGGCCTACAGA-3′, reverse 5’-GGGAGTTCTCAGCCTCCAG-3′; S100*β*, forward 5’-GAAGGGAGGGAGACAAGCAC-3′, reverse 5’-CCTCCTGCTCTTTGATTTCCTCT-3′. All primers pairs were designed to overlap exon-exon junctions to avoid contamination signal from genomic DNA. Samples were analyzed in duplicates. Relative gene expression was quantified using the comparative ΔΔ Ct method and normalized to β-actin and Cyclophilin A transcript levels (Livak and Schmittgen, 2001).

### Brain organoid treatment

Brain organoids were maintained in DMEM medium (Merck) supplemented with 25 mM Glucose for one to two days before treatment. For treatment, medium was switched to stimulation medium for 10-min duration during which electrical signal was recorded. For experiments involving activity modulation, stimulation medium consisted in DMEM +25 mM glucose that was supplemented with Veh (0.1% dimethylsulfoxide, DMSO), potassium chloride (KCl, 5 mM; Merck) or tetrodotoxin (TTX, 200 nM; Abcam). For analysis of electrical activity under different glucose concentrations, organoids were treated for 10 min with DMEM supplemented with 25 mM glucose, which served as activity baseline for each organoid, followed by treatment for 10 min with DMEM supplemented with 5 mM glucose.

### Microelectrode array, electrophysiology platform and electrical signal recording

**Figure 1:**
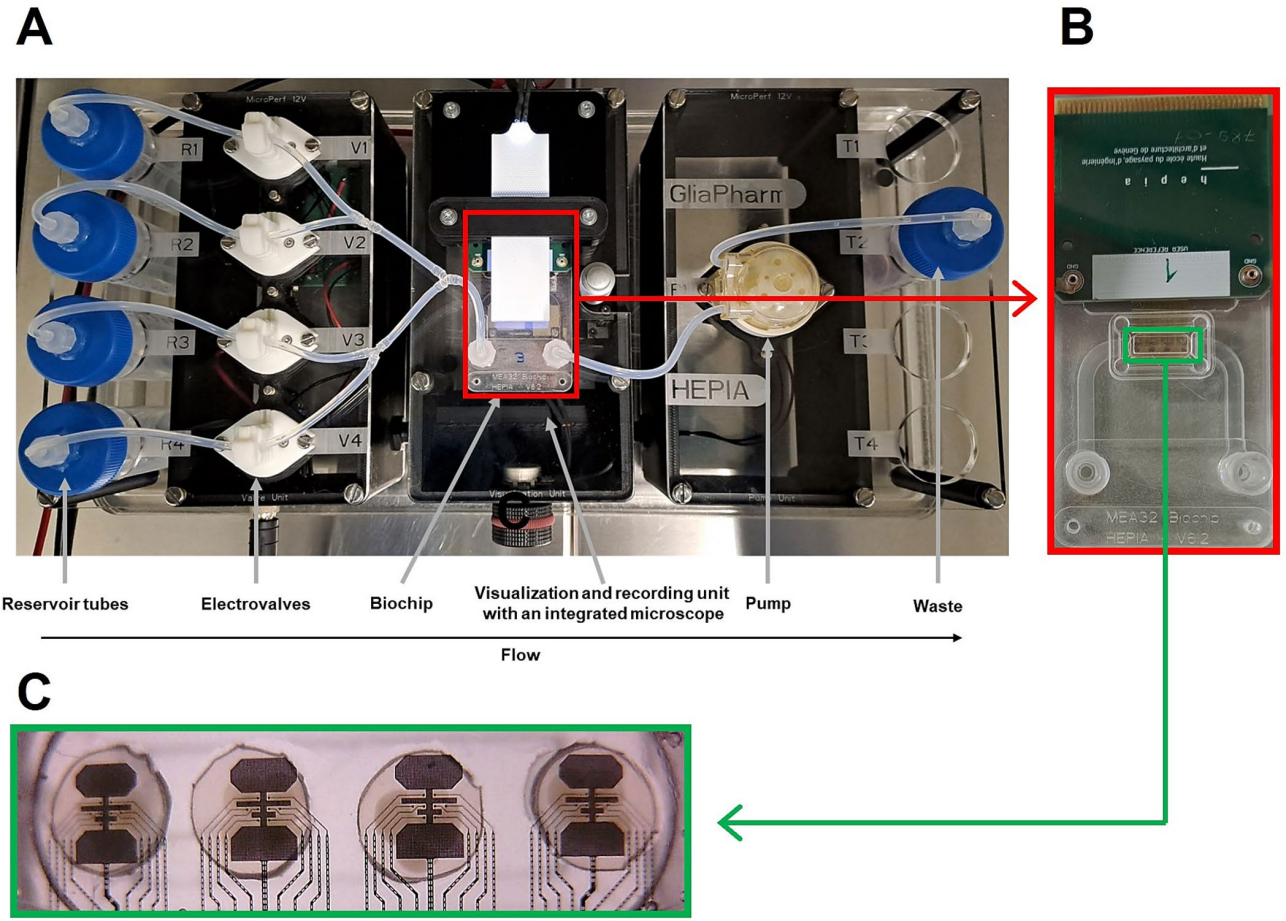
Custom-made electrophysiology platform for recording brain organoids’ activity (A), zoomed in view of a biochip shown in red rectangle (B), zoomed in view of the 4 micro-electrode arrays (MEAs) in green rectangle (C).

At week 7, brain organoids were placed to an air liquid interface (ALI) culture as described previously (Govindan et al., 2020). After two days on ALI membranes, four brain organoids were placed on recording sites of a custom-made Microelectrode array (MEA) biochip (Wertenbroek et al., 2021) for a minimum of 5 days for proper adherence of the organoid to the biochip. The electrophysiology platform consisted of four reservoirs, 4 electrovalves, a peristaltic pump as well as visualization and recording unit with integrated camera (Figure 1A). After 5 days of culture, the biochip (Figure 1B) was connected to the custom-made electrophysiology platform housed in an incubator at 37°C and 5% CO_2_, featuring an automated flow pumping system with perfusion rate of 0.53 mL/min for remote media changes. The electrical signal of each electrodes of the biochip (Figure 1C) was recorded in real time using SpikeOnChip (SPOC) acquisition system (Govindan et al., 2020). The biochip consisted of a fluidic chamber which central part was open to the ALI via a porous polyimide membrane of 8 μm thickness, with 5 μm holes and 10% surface porosity. 150 nm-thick platinum wires and platinum coated electrodes of 30 μm diameter were incorporated into the membranes. No additional coating was applied to the electrodes prior to organoid deposition. Each biochip was composed of four recording sites, each of which made of eight recording electrodes and three reference electrodes.

### Electrophysiology processing

Electrical signals from the SpikeOnChip (SPOC) acquisition system were detected within 0.1 Hz to 15 kHz frequency range. A RHD2132 Digital Electrophysiology interface chip (Intan Technologies) was used to amplify and convert voltage from the 32 electrodes of the biochip into digital data. The RHD2132 chip allowed a sampling rate of 30 kS/s with a resolution of 16 bits. The signal was amplified from 0.1 Hz to 5 kHz. The digital data was sent to a FPGA electronic card, which generated three types of data: Unprocessed raw data, spike window cut-outs, and Local Field Potential Analysis (LFPA) values. Data were processed by an ARM processing system (PS) and transmitted to the host computer running the GUI software SPOC (Wertenbroek et al., 2021). Spike cut-outs were automatically selected as 5 ms events when the signal was above a threshold set at 6 times the standard deviation from electrical activity’s baseline (Govindan et al., 2020). A custom Python script was employed to detect bursts of spikes, defined as a series of at least five consecutive spikes with a maximal interval of 50 ms between each of them (Bakkum et al., 2013).

### Epileptiform discharges detection

Epileptiform discharges detection was performed using custom Python scripts. The raw signals were first preprocessed to remove noise and isolate relevant neural activity. A 50 Hz notch filter, implemented using a second-order infinite impulse response (IIR) filter (iirnotch function from the *scipy.signal* library) with a quality factor of 30, was applied to eliminate electrical line noise interference. This ensured attenuation of the 50 Hz frequency component without affecting neighboring frequencies. Subsequently, a second-order Chebyshev Type I bandpass filter with a frequency range of 4 to 40 Hz was use to further process the signal. This filter was chosen to encompass the typical frequency bands of interictal epileptic spikes and sharp waves observed in both human and animal studies (Heining et al., 2019; Pillai and Sperling, 2006; Smith et al., 2022). The Chebyshev Type I filter, designed with a 0.5 dB passband ripple, was implemented using the *cheby1* function from *scipy.signal* and applied with zero-phase filtering (*filtfilt function*) to preserve spike timing integrity. Epileptiform discharges were then identified using a threshold-based approach, where discharges were defined as events exceeding the mean amplitude of the filtered signal by more than 4.5 standard deviations. To prevent the detection of multiple discharges originating from the same event, a refractory period of 50 ms was applied. This refractory period is critical, as a single epileptiform event can cause multiple peaks or fluctuations that exceed the detection threshold I quick succession, which, without the refractory period, would be mistakenly classified as independent events. A time-frequency representation of the signal was also generated to visualize neural activity patterns. To reduce computational complexity, the raw signal, initially sampled at 30 kHz, was downsampled to 100 Hz using the decimate function from the *scipy.signal* package. This downsampling was sufficient to capture relevant neural activity while improving data processing efficiency. The Morlet wavelet transform was applied to calculate the corresponding time-frequency scales within the specified frequency limits. The continuous wavelet transform (CWT) was computed to obtain a detailed time-frequency representation of the event. The power (dB) of the wavelet coefficients was normalized relative to the baseline period to highlight significant trains of electrical signal. Specifically, the mean and standard deviation of the baseline power were used to normalize the power values into z-scores. Any values below two standard deviations from the baseline were set to a minimum threshold to focus on the most significant frequencies. These normalized power plots, visualized on a logarithmic scale, provided a comprehensive overview of the frequency components associated with epileptiform discharges.

### Statistical analyses

Statistical significance was calculated using unpaired two-tailed Student’s t-test, non-parametric Mann Whitney test when homoscedasticity and/or normal distribution were not met, one-way analysis of variance (ANOVA) followed by Bonferroni’s post-hoc test or Kruskal-Wallis non-parametric ANOVA followed by Dunn’s multiple comparison test when homoscedasticity and/or normal distribution were not met. Outliers were detected using ROUT’s test and removed from the analysis. GraphPad Prism v10 was used for all statistical analyses. *, ** and *** refer to *p* values of <0.05, 0.01 and 0.001, respectively. The total number of replicates (n) is indicated in the figure legends.

## Results

### Brain organoids cellular composition

**Figure 2:**
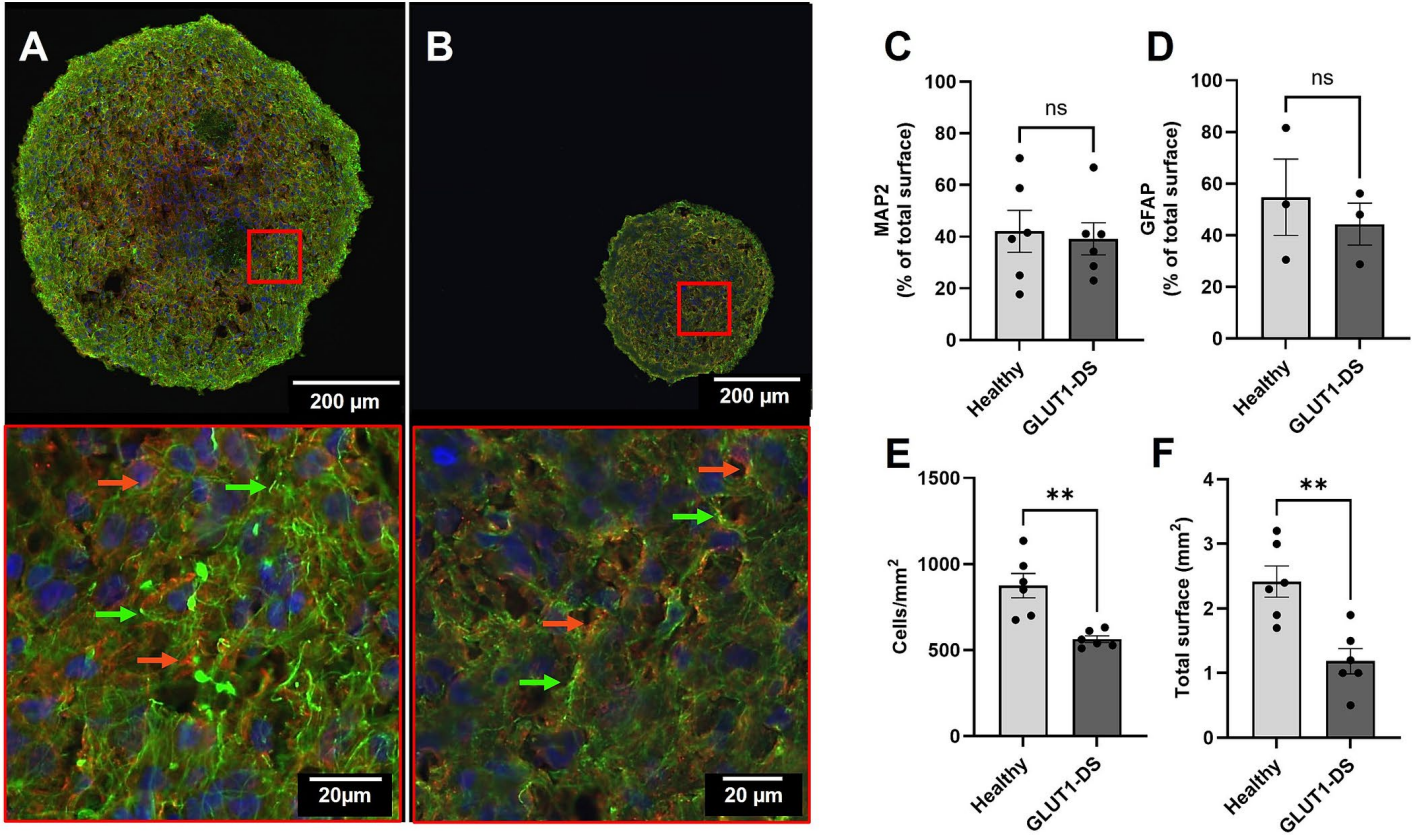
Immunostaining of healthy and GLUT1-DS brain organoids. (A,B) Representative immunostaining images of brain organoids derived from healthy individual (A) and GLUT1-DS patient (B), showing Glial Fibrillary Acidic Protein (GFAP; green) for astrocytes, Microtubule Associated Protein 2 (MAP2; red) for neurons, and DAPI (blue) for nuclei. Green and red arrows point to astrocytes and neurons, respectively. (C,D) Quantification of the surface occupied by MAP2 (C) and GFAP (D), shown as the average percentage ± S.E.M of total surface of the organoid (*n* = 3–6). (E) Cellular density, expressed as the number of DAPI+ cell nuclei per mm^2^ (*n* = 6). (F) Total area of brain organoids (mm^2^) (*n* = 6). Statistical analyses were performed using unpaired bilateral Student’s t-test (ns, not significant; ** *p* < 0.01).

Brain organoids derived from iPSCs from both healthy individual and GLUT1-DS patient were cultured to maturity over a period of 12 weeks (Govindan et al., 2020). The mutation in GLUT1 is characterized by a proline-to-leucine change (P485L), which leads to formation of dileucine motifs that result in protein mistrafficking (Meyer et al., 2018). Upon reaching maturity, we quantified cellular composition, density and size using immunostaining for the astrocytic marker GFAP and the neuronal marker MAP2 (Figures 2A,B). Our results confirmed the presence of both cell types, with no differences in the area labelled by each marker between healthy and GLUT1-DS organoids, as quantified by the percentage of total surface (Figures 2B–D). Despite similar marker distribution, our data showed that cellular density, as measured by DAPI nuclei staining, was significantly reduced in GLUT1-DS organoids (Figure 2E). Total organoid area was also smaller in GLUT1-DS-derived cultures compared to healthy ones (Figure 2F). These findings indicate that while astrocytes and neurons are similarly expressed in both healthy and GLUT1-DS organoids, overall cellular density and organoid size are decreased in GLUT1-DS organoids.

### iPSCs and brain organoids mRNA expression profiles

**Figure 3:**
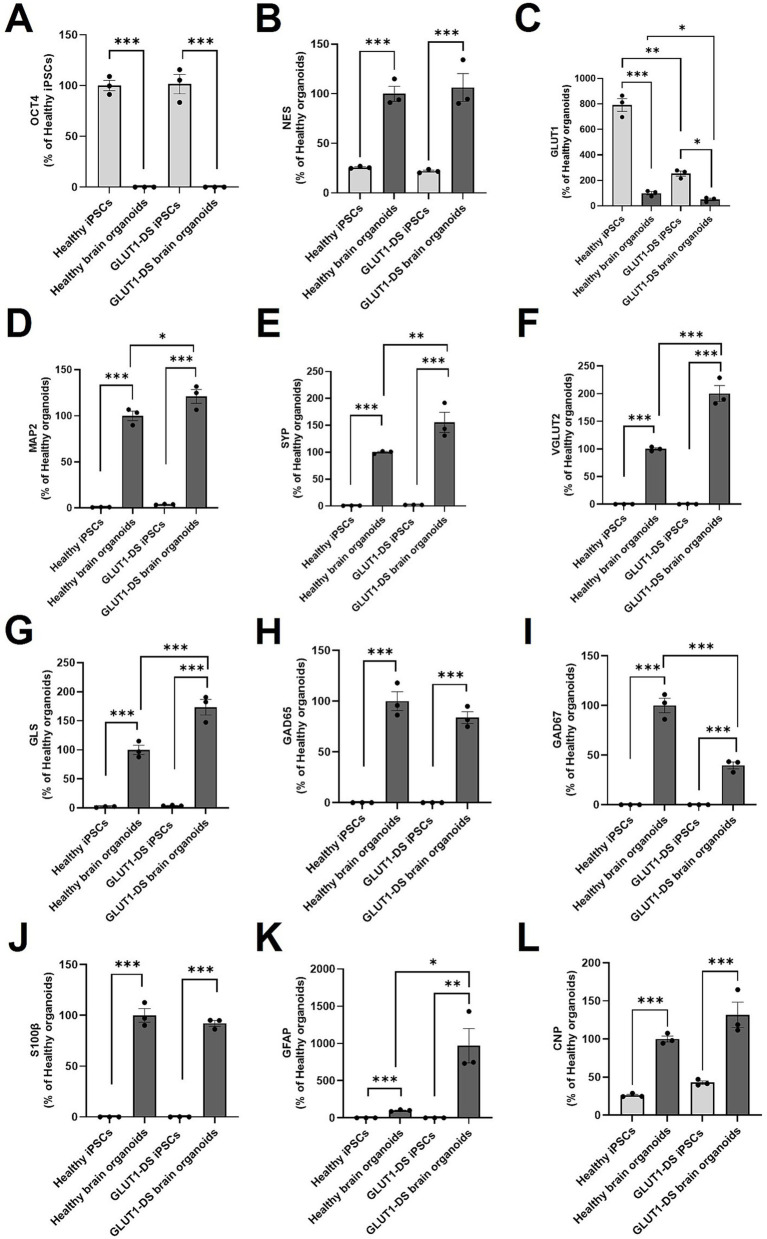
mRNA expression in healthy and GLUT1-DS iPSCs and brain organoids. mRNA expression levels were quantified by Real-Time qPCR for the following markers: (A) OCT4, a stem cell marker; (B) Nestin (NES), a marker for neural stem cells and axonal growth; (C) glucose transporter 1 (GLUT1); (D) microtubule associated protein 2 (MAP2), a marker of neuronal cytoskeleton; (E) synaptophysin (SYN), a synaptic marker; (F) vesicular glutamate transporter 2 (VGLUT2), a marker of glutamatergic neurons; (G) glutaminase (GLS), another marker of glutamatergic neurons; (H) glutamate decarboxylase 65 (GAD65) and (I) glutamate decarboxylase 67 (GAD67), two markers of GABAergic neurons; (J) S100β marker for astrocytes; (K) Glial Fibrillary Acidic Protein (GFAP), a marker of mature and reactive astrocytes; (L) 2′,3′-cyclic nucleotide 3′-phosphodiesterase (CNP), a marker of developing oligodendrocytes. mRNA expression levels were normalized to Actin and Cyclophilin levels and presented as percentage changes relative to healthy organoids levels, except in (A) where this was presented as percentage changes relative to healthy iPSCs levels because there was no expression of OCT4 in organoids. Statistical analyses were performed using one-way ANOVA followed by Bonferroni’s post-hoc tests (*n* = 3, * *p* < 0.05, ** *p* < 0.01, *** *p* < 0.001).

Next, we evaluated the cellular maturity of healthy and GLUT1-DS brain organoids by gene expression using RT qPCR. Organoids were grown for 2 months, a developmental period that has been shown to support the cellular maturation of the different cell types and to produce functional neural networks with stable and reproducible electrophysiological signal (Porciuncula et al., 2021). As expected, expression of OCT4, a marker of pluripotent stem cells, was markedly reduced in organoids from both origins compared to respective iPSCs (Figure 3A). Conversely, Nestin (NES), a marker for cellular maturation and axonal growth was significantly increased in organoids compared to iPSCs (Figure 3B). The reduction of OCT4 and the increase in NES confirm the maturation of both types of organoids. Interestingly, expression of GLUT1 was elevated in iPSCs compared to organoids, while levels were significantly lower in GLUT1-DS than healthy iPSCs and organoids (Figure 3C). Additionally, neuronal markers such as MAP2, Synaptophysin (SYP), vesicular glutamate transporter 2 (VGLUT2), glutaminase (GLS), glutamate decarboxylase 65 and 67 (GAD65, GAD67) were all elevated in both organoid types compared to the iPSCs (Figures 3D–G). Data indicate that MAP2, SYP, VGLUT2 and GLS were significantly more expressed in GLUT1-DS organoids compared to healthy ones (Figures 3D–G), while expressions of GABAergic neuronal markers GAD65 and GAD67 were reduced in GLUT1-DS organoids compared to healthy ones (Figures 3H,I). Markers for astrocytes, including S100β and GFAP, were also upregulated in both healthy and GLUT1-DS organoids relative to iPSCs. While there was no significant difference in S100β expression between healthy and GLUT1-DS organoids (Figure 3J), a marked increase in GFAP expression was observed in GLUT1-DS organoids compared to healthy ones (Figure 3K). Finally, the expression of 2′,3′-cyclic nucleotide 3′-phosphodiesterase (CNP), a marker for developing oligodendrocytes, was elevated in organoids from both origins compared to iPSCs, with no significant differences between the two types of organoids (Figure 3L).

### Neuronal activity in brain organoids

**Figure 4:**
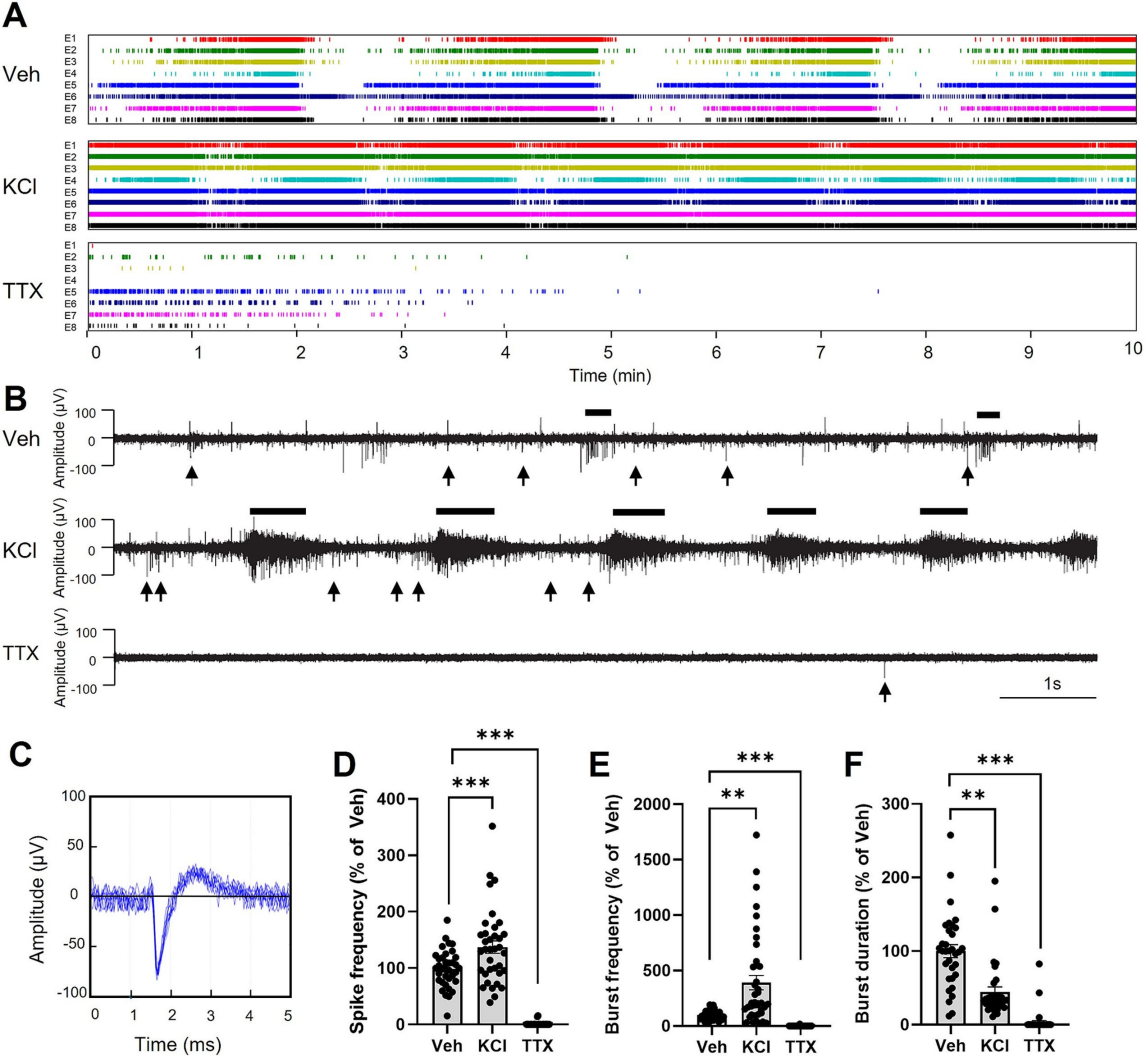
Modulation of electrical signal in brain organoids. (A) Examples of raster plots of spikes detected by the SPOC system from 8 electrodes (E1-E8) for each brain organoid. Organoids were treated for 10 min with vehicle (Veh), potassium chloride (KCl; 5 mM added to the existing 5 mM in the culture medium; total 10 mM), or tetrodotoxin (TTX; 200 nM). (B) Representative neuronal activities from brain organoids in each condition. Black lines above each graph highlight examples of bursts, while black arrows below each graph indicate selected instance of individual spikes that do not contribute to burst formation. (C) Representation of the shape (amplitude and duration) of a typical spike detected in (A,B), shown as the superposition of 10 consecutive spikes. (D–F) Quantification of the spike frequency (D), bursts frequency (E) and burst duration (F), during the first 2 min of recording. Results are presented as the mean ± SEM of percentage change compared to vehicle (*n* = 33 to 40 electrodes from 7 healthy brain organoids). Statistical analyses were performed using Kruskal-Wallis non-parametric ANOVA test followed by Dunn’s multiple comparison test (** *p* < 0.01, *** *p* < 0.001).

We then recorded electrical signal using MEA in brain organoids across frequencies ranging from 0.1 Hz to 15 kHz. Spikes were automatically detected by the SPOC acquisition software (Govindan et al., 2020), as shown here as raster plots for each electrode (8 electrodes per MEA; Figure 4A). Examples of typical neuronal activity for each condition and spike waveform as recorded are shown (Figures 4B,C, respectively). To modulate electrical signals in brain organoids, we treated them with potassium chloride (KCl) for 10 min to induce membrane depolarization and trigger spike generation (Gorji et al., 2003). Our data show that KCl treatment significantly increased both spike and burst frequencies while decreasing the burst average duration within the first 2 min of application (Figures 4D–F). Bursts were defined as a minimum of 5 consecutive spikes with maximum interval of 50 ms between them. In contrast, the addition of tetrodotoxin (TTX), which aimed at blocking voltage-gated sodium channels (Dubois-Dauphin et al., 2010), suppressed neuronal activity, as indicated by the reduced spike frequency, burst frequency and bursts duration, like shown in previous studies (Stett et al., 2003) (Figures 4D–F). All stimulations described here were performed on healthy brain organoids in culture medium containing 25 mM glucose. These findings indicate that the brain organoids exhibit a mature electrical profile, which can be quantified through spike and burst parameters, and can be modulated by agents such as KCl and TTX.

### Epileptiform activity in healthy and GLUT1-DS brain organoids

**Figure 5:**
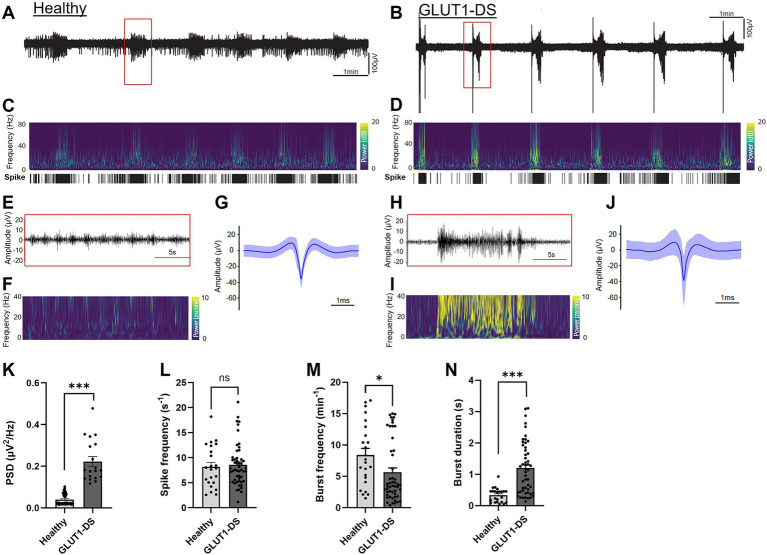
Epileptiform activity in healthy and GLUT1-DS brain organoids (A,B) Neuronal activity (0.1 Hz-15 kHz) in brain organoids from healthy (A) and GLUT1-DS origins (B), cultured in medium containing 25 mM glucose. (C,D) Time frequency plots and spike mappings in healthy (C) and GLUT1-DS (D) brain organoids, corresponding to the signal shown A,B, respectively. (E,F) Zoomed in view of the activity (E) and power (F) of a burst from a healthy brain organoid highlighted with a red rectangle in A, within filtered frequency range (4–40 Hz). (G) Average spike waveforms (0.1 Hz-15 kHz; *n* = 3,166) from a healthy brain organoid during a 10-min recording period. (H,I) Zoomed in view of the activity (H) and power (I) of a burst from a GLUT1-DS organoid highlighted with a red rectangle in B, within 4–40 Hz filtered frequency range. (J) Average spike waveforms (0.1 Hz-15 kHz; *n* = 4,670) from a GLUT1-DS brain organoids during a 10-min recording period. (K) Average bursts’ power spectrum density (PSD) within 4–40 Hz filtered frequency range during a 10-min recording period in healthy and GLUT1-DS brain organoids (*n* = 19–48). (L–N) Average spike frequency (L), burst frequency (M) and burst duration (N) during a 10-min recording period in healthy and GLUT1-DS brain organoids (*n* = 23–50). Data are presented as the mean + SEM. Statistical analyses were performed using Mann–Whitney non-parametric test (ns, not significant, * *p* < 0.05, *** *p* < 0.001).

We further analyzed neuronal activity in healthy and GLUT1-DS brain organoids by detection of spikes and bursts of spikes within the 0.1 Hz to 15 kHz frequency range. Representative examples of activity from healthy and GLUT1-DS brain organoids in culture medium containing 25 mM glucose are shown (Figures 5A,B, respectively). Time frequency plot analyses indicate lower power in brain organoids from healthy origin (Figure 5C) compared to GLUT1-DS ones (Figure 5D). Importantly, our data indicates that bursts of spikes, as detected within the 0.1 Hz to 15 kHz frequency range and shown in Figures 5A–D, co-occur with activity at lower frequency range (filtered at 4–40 Hz), where epileptiform discharges are commonly observed (Heining et al., 2019; Padmasola et al., 2024). Time frequency plots show that power is lower in healthy brain organoids (Figure 5F) than in GLUT1-DS brain organoids (Figure 5I) within this frequency range, indicative of epileptiform feature. Further, aggregates of spikes pattern (0.1–15 kHz) during 10-min recording for healthy (Figure 5G) and GLUT1-DS organoids (Figure 5J) indicate that spikes have larger amplitudes in GLUT1-DS brain organoids compared to healthy ones (Figures 5G,J). Power spectrum density (PSD) was quantified for each burst detected during recording within 4–40 Hz frequency range, and indicates a significantly higher bursts PSD in GLUT1-DS brain organoid compared to healthy ones (Figure 5K), which, within this frequency range, is indicative of epileptiform activity. While no differences in spike frequency were observed between healthy and GLUT1-DS brain organoids (Figure 5L), the bursts pattern differed between the two types of organoids. Specifically, GLUT1-DS brain organoids exhibited a lower bursts frequency (Figure 5M) but longer bursts duration (Figure 5L) compared to healthy organoids. Together, these findings demonstrate distinct differences in neuronal activity between healthy and GLUT1-DS brain organoids, which can be quantified through spike detection (0.1 Hz – 15 kHz frequency range). These differences are characteristics of epileptiform discharges in GLUT1-DS organoids, as shown by the increased power density in the filtered 4–40 Hz frequency range.

### Increased neuronal activity in GLUT1-DS exposed to lower glucose concentrations

**Figure 6:**
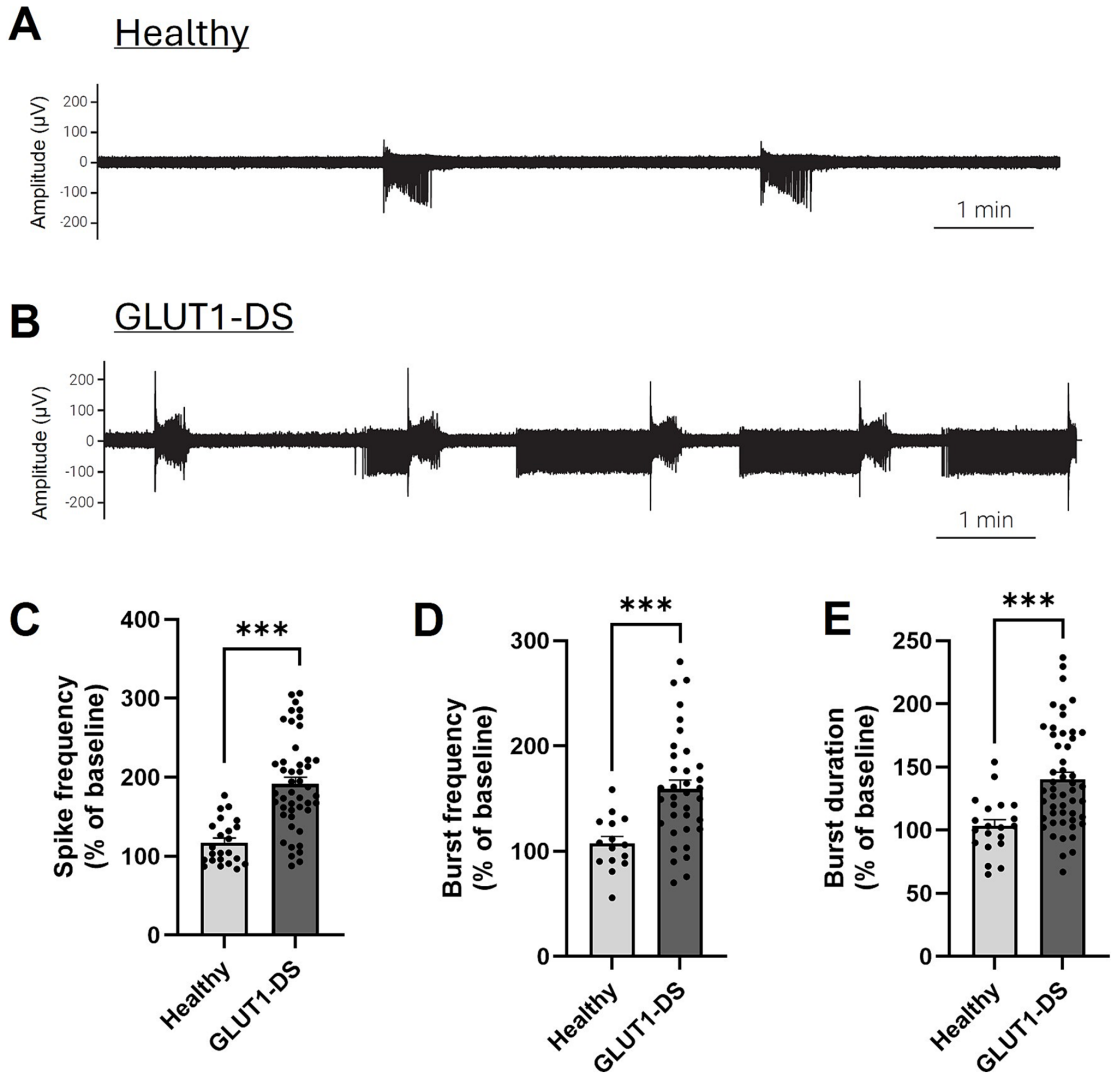
Increased activity in GLUT1-DS brain organoids under low glucose conditions. (A,B) Representative neuronal activity (0.1 Hz-15 kHz) in brain organoids from healthy (A) and GLUT1-DS (B) origins, cultured in medium containing 5 mM glucose. (C) Spike frequency (0.1 Hz – 15 kHz) measured for 10 min in medium containing 5 mM glucose, represented as the percentage change compared to baseline in 25 mM glucose (*n* = 24–45 electrodes from 6 organoids of each type). (D,E) Frequency (D) and duration (E) of bursts (0.1 Hz – 15 kHz) measured for 10 min in medium containing 5 mM glucose, represented as the percentage change compared to baseline in 25 mM glucose (*n* = 20–50 and *n* = 12–34 electrodes for D,E, respectively, from 6 organoids of each type). Data are presented as the mean + SEM. Statistical analyses were performed using Mann–Whitney non-parametric test (*** *p* < 0.001).

Finally, we recorded neuronal activity in brain organoids from healthy and GLUT1-DS origins for 10 min in medium containing 25 mM glucose, followed by a 10-min recording period in medium with 5 mM glucose concentration. Neuronal activity in 25 mM glucose concentrations served as the baseline for each organoid, and activity in 5 mM glucose was compared against this baseline (Figure 6). Neuronal activity pattern in healthy and GLUT1-DS brain organoids exposed to 5 mM glucose for 10 min is shown (Figures 6A,B). Spikes were detected within 0.1 Hz to 15 kHz frequency range during the 10-min recording periods (Figure 6C), while bursts were defined as a series of least 5 spikes with maximum intervals of 50 ms between them (Figures 6D,E). When comparing neuronal activity of GLUT1-DS organoids to healthy ones in 5 mM glucose, we observed increased frequency of spikes (Figure 6C), bursts frequency (Figure 6D) and burst duration (Figure 6E) in GLUT1-DS brain organoids. Thus, our results show that neuronal activity in GLUT1-DS organoids significantly increased when exposed to lower glucose concentrations (5 mM) compared to baseline (25 mM glucose), whereas the activity of healthy organoids remained unchanged under reduced glucose conditions. These findings indicate a highlighted neuronal excitability of GLUT1-DS brain organoids to decreased glucose availability, consistent with epileptiform pattern.

## Discussion

GLUT1-DS is a rare genetic condition characterized by impaired glucose uptake in the brain, resulting in hypoglycorrhachia and neurological symptoms that include epileptic seizures from infancy. Although transgenic mouse models can recapitulate deficit in the expression of GLUT1 and brain hypometabolism (Furuse et al., 2019; Tang and Monani, 2021; Tang et al., 2021; Wang et al., 2006), monitoring epilepsy in these models has proven challenging. Moreover, differences in species-specific brain architecture, metabolism, and developmental processes can sometimes limit the translatability of findings to human conditions. This limitation underlines the growing interest in using human-derived organoids as a complementary approach to study pathologies. Brain organoids are becoming an increasingly valuable tool for the screening and testing of therapeutic strategies, including drug discovery and development, offering a fast and efficient alternative to traditional animal models (Amin and Pasca, 2018; Di Lullo and Kriegstein, 2017; Qian et al., 2019). In this study, we utilized human brain organoids derived from iPSCs from a healthy individual and a GLUT1-DS patient to monitor structural and electrical activity, with a particular focus on epileptiform patterns.

Initially, we demonstrated that human brain organoids could be successfully developed from iPSCs of healthy and GLUT1-DS origins. These organoids predominantly consist of neurons and astrocytes, with no significant difference in the spatial distribution of these cell types (Figure 2). However, organoids derived from GLUT1-DS patients exhibited a marked reduction in cellular density and total size (Figures 2E,F). This finding aligns with microcephaly observed in GLUT1-DS patients, which is attributable to the characteristic hypometabolism during brain development (Pascual et al., 2002; Pascual et al., 2007; Wang et al., 2006). It is to note that the brain organoids do not include endothelial cells, as these cells do not originate from neural stem cells. Given that endothelial cells also express GLUT1, they play a significant role in the reduced brain glucose uptake observed in GLUT1-DS pathology (Tang et al., 2017). Therefore, our study focuses on the specific role of diminished GLUT1 in astrocytes, leading to impaired metabolism and abnormal neuronal activity in GLUT1-DS. Future studies incorporating endothelial cells into brain organoids to form functional capillary networks would be required to elucidate the impact of endothelial GLUT1 deficiency in neuronal function.

The mRNA expression profiles confirmed the maturation of the organoids relative to the iSPCS, as evidenced by a significant reduction of pluripotent stem cell marker OCT4 in both organoid types, along with increased expression of markers associated with axonal development (Nestin), neuronal maturity (MAP2, Synaptophysin, VGLUT2, Glutaminase, GAD65, GAD57), and astrocytes maturity (S100β and GFAP) (Figure 3). Notably, when comparing brain organoids from both groups, the GLUT1-DS organoids showed elevated expression of several genes, including MAP2 and GFAP. Despite this, immunostaining revealed no difference in spatial distribution of MAP2 and GFAP, while overall cells number was reduced (Figure 2), suggesting a reorganization of cytoskeletal architecture in GLUT1-DS organoids, with more territory occupied by each cell types. Interestingly, the increased GFAP expression in astrocytes may be indicative of reactive phenotype and morphological changes often associated with inflammatory conditions in neurological diseases, such as Alzheimer’s disease and amyotrophic lateral sclerosis (ALS) (Benninger et al., 2016; Chatterjee et al., 2021; Cicognola et al., 2021; Yeh et al., 2011). Additionally, qPCR data reveal an increased expression of excitatory glutamatergic neuron-specific markers (VGLUT2, glutaminase) in GLUT1-DS brain organoids, while expression of inhibitory GABAergic neuron-specific markers (GAD65, GAD67) were reduced compared to healthy brain organoids (Figure 3). This shift in the excitatory-inhibitory balance may account for the heightened excitability in GLUT1-DS brain organoids (Sarlo and Holton, 2021). Moreover, qPCR analysis shows a significant reduction in GLUT1 expression in organoids compared to iPSCs, with a more pronounced decrease in GLUT1-DS, both in iPSCs and organoids (Figure 3C). While the GLUT1 mutation (P485L) is known to affect its trafficking (Meyer et al., 2018), the observed reduction in GLUT1 gene expression may be a secondary consequence of the transporter’s impaired functionality.

We further assessed neuronal activity in organoids by quantifying spikes frequency using microelectrode array (MEA) recording at frequencies between 0.1 Hz and 15 kHz. The experimental setup involved culturing brain organoids on an air-liquid interface (ALI), which was shown to be essential for correct oxygenation of the tissue over extended periods of time (Stoppini et al., 2024). A custom-made pumping system allowed for seamless media changes and continuous recording of electrical activity on biochips (Wertenbroek et al., 2021). Within the recorded frequency range, spikes were clearly identifiable, characterized by signals exceeding six times standard deviation of baseline electrical activity, typically reaching 100 μV amplitude. Repetitive spikes formed bursts, defined by a minimum of five repeated spikes with intervals of at least 50 ms between them. Our data indicate that the addition of potassium chloride (KCl; 5 mM, on top of the existing 5 mM in the culture medium) to depolarize neuronal membranes resulted in a significant increase in the number of spikes, as well as an increase in burst frequency and a decrease in burst duration over a two-minute recording period (Figure 4). Conversely, the addition of tetrodotoxin (TTX), which aims at blocking voltage-dependent Na^+^ channels thus inhibiting neuronal depolarization, led to a marked reduction in the number of spikes, bursts, and burst durations (Figure 4). These findings confirm that the organoids used in this study exhibited functional neuronal activity.

The comprehensive analysis of electrical activity within the 0.1 Hz to 15 kHz range, while broad, was crucial for accurately identifying spikes, which predominantly occur within the 600 Hz to 6,000 Hz range. Further analysis revealed that signals in this broad frequency band were associated with epileptiform discharges that are occurring in the lower frequency range of 4 to 40 Hz, though these latter exhibited lower amplitudes. The lower amplitude of the epileptiform discharges compared to spikes is likely explained by the fact that brain organoids lack the neuronal geometry and architecture that is essential in shaping the amplitude of epileptiform discharges (Buzsaki et al., 2012; Thio and Grill, 2023). Furthermore, as our approach focused on capturing overall network activity rather than isolating individual neuronal units, no spike waveform sorting was performed, which may result in the detection of signals from more than one neuron in a single electrode and could influence the observed spike characteristic. Thus, while our primary analysis focused on a broad frequency range (0.5 Hz-15 kHz) with better detection of electrical signal, the co-occurring pattern of activity at low frequency ranges (4–40 Hz) aligned with epileptiform activity. Notably, neuronal activity in the 4 to 40 Hz range displayed characteristic epileptiform discharges in GLUT1-DS brain organoids, as evidenced by the time-frequency plots and strong power signals typical of epileptic activity (Figures 5F,I,K).

Next, we examined how varying glucose concentrations in the culture medium affected neuronal activity in both healthy and GLUT1-DS brain organoids. Our results showed that reducing the extracellular glucose concentration from 25 mM (baseline) to 5 mM for 10 min did not significantly alter the neuronal activity of healthy organoids, while it markedly increased that of GLUT1-DS organoids (Figure 6). These data indicate that GLUT1-DS brain organoids are more sensitive to metabolic stress than healthy ones, leading to an increased number of spikes, higher burst frequency, and prolonged burst durations, consistent with an epileptiform activity.

In summary, our study validated the methods for developing human brain organoids from iPSCs derived from GLUT1-DS patients and demonstrated that these organoids exhibit epileptiform features when compared to those developed from healthy iPSCs. This novel technology could help identifying and developing new therapeutic strategies aimed at improving glucose metabolism in GLUT1-DS. This includes our own ongoing efforts to develop innovative treatments targeting astrocytic glycolysis to enhance glucose uptake and metabolism in hypometabolic neurological conditions like GLUT1-DS (Beard et al., 2021; Finsterwald et al., 2015). Furthermore, this technology could open new avenues for research and development of therapeutic approaches in other genetic-based rare epilepsies by enabling the analysis of different epileptic profiles in patient-specific brain organoids.

## Data Availability

The original contributions presented in the study are included in the article, further inquiries can be directed to the corresponding author.
